# Genetic and geographic influence on phenotypic variation in European sarcoidosis patients

**DOI:** 10.3389/fmed.2023.1218106

**Published:** 2023-08-09

**Authors:** Sandra Freitag-Wolf, Jonas C. Schupp, Björn C. Frye, Annegret Fischer, Raihanatul Anwar, Robert Kieszko, Violeta Mihailović-Vučinić, Janusz Milanowski, Dragana Jovanovic, Gernot Zissel, Elena Bargagli, Paola Rottoli, Dragos Bumbacea, René Jonkers, Ling-Pei Ho, Karoline I. Gaede, Anna Dubaniewicz, Ben G. Marshall, Andreas Günther, Martin Petrek, Michael P. Keane, Sigridur O. Haraldsdottir, Francesco Bonella, Christian Grah, Tatjana Peroš-Golubičić, Zamir Kadija, Stefan Pabst, Christian Grohé, János Strausz, Martina Safrankova, Ann Millar, Jiří Homolka, Wim A. Wuyts, Lisa G. Spencer, Michael Pfeifer, Dominique Valeyre, Venerino Poletti, Hubertus Wirtz, Antje Prasse, Stefan Schreiber, Astrid Dempfle, Joachim Müller-Quernheim

**Affiliations:** ^1^Institute of Medical Informatics and Statistics, Kiel University, Kiel, Germany; ^2^Department of Pneumology, Faculty of Medicine, University Medical Centre, Freiburg, Germany; ^3^Department of Respiratory Medicine, Hannover Medical School, German Center for Lung Research (DZL), Hannover, Germany; ^4^Biomedical Research in End-Stage and Obstructive Lung Disease (BREATH), Hannover Medical School (MHH), German Center for Lung Research (DZL), Hannover, Germany; ^5^Institute of Clinical Molecular Biology, Kiel University, Kiel, Germany; ^6^Department of Pneumonology, Oncology and Allergology, Medical University of Lublin, Lublin, Poland; ^7^Department of Pneumology, University Hospital, Belgrade, Serbia; ^8^Respiratory Diseases and Lung Transplant Unit, University Hospital, Siena, Italy; ^9^Department of Cardio-Thoracic Medicine, Carol Davila University of Medicine and Pharmacy, Bucharest, Romania; ^10^Pulmonology Department, Academic Medical Center Amsterdam, Amsterdam, Netherlands; ^11^Oxford Sarcoidosis Service, Oxford University Hospitals NHS Foundation Trust, Churchill Hospital, Oxford, United Kingdom; ^12^Medical Hospital, Research Center Borstel, Borstel, Germany; ^13^Department of Pulmonology, Medical University of Gdansk, Gdansk, Poland; ^14^Department of Respiratory Medicine, University Hospital, Southampton, United Kingdom; ^15^Department of Pneumology and Intensive Care, University Hospital, Giessen, Germany; ^16^Faculty of Medicine and Dentistry, Palacký University and University Hospital Olomouc, Olomouc, Czechia; ^17^Division of Pulmonary and Critical Care Medicine, University College Dublin and St Vincent’s University Hospital, Dublin, Ireland; ^18^Landspitali, University Hospital, Reykjavik, Iceland; ^19^Ruhrlandklinik, Westdeutsches Lungenzentrum am Universitätsklinikum Essen, Universitätsklinik Essen, Essen, Germany; ^20^Hospital Berlin-Havelhöhe, Berlin, Germany; ^21^Pulmonary Department, University Hospital Jordanovac, Zagreb, Croatia; ^22^Foundation IRCCS Policlinico San Matteo - Pulmonology Unit, Pavia, Italy; ^23^Department of Pneumology, University Hospital, Bonn, Germany; ^24^Evangelische Lungenklinik Berlin, Berlin, Germany; ^25^National Koranyi Institute, Budapest, Hungary; ^26^Thomayer Hospital and 1st Faculty of Medicine, Charles University, Praha, Czechia; ^27^Pulmonary Department, University Hospital, Bristol, United Kingdom; ^28^Prague General Hospital, Charles University, Prague, Czechia; ^29^Laboratory of Respiratory Diseases and Thoracic Surgery (BREATHE), University Hospital, Leuven, Belgium; ^30^Liverpool Interstitial Lung Disease Service, Aintree Chest Centre, Liverpool University Hospitals NHS FT, Liverpool, United Kingdom; ^31^Department of Pneumology, University Hospital Regensburg, Regensburg, Germany; ^32^Groupe Hospitalier Avicenne-Jean Verdier-René Muret, Service de Pneumologie, Bobigny, France; ^33^Pulmonary Unit, Department of Thoracic Diseases, Azienda USL Romagna, GB Morgagni-L-Pierantoni Hospital, Forlì, Italy; ^34^Department of Pneumology, University Hospital Leipzig, Leipzig, Germany; ^35^Department of Internal Medicine I, University Hospital Schleswig-Holstein, Kiel, Germany

**Keywords:** genetic polymorphism, genetic risk factors, sarcoidosis, genotype–phenotype-relationship, region-specific genetic links

## Abstract

**Introduction:**

Sarcoidosis is a highly variable disease in terms of organ involvement, type of onset and course. Associations of genetic polymorphisms with sarcoidosis phenotypes have been observed and suggest genetic signatures.

**Methods:**

After obtaining a positive vote of the competent ethics committee we genotyped 1909 patients of the deeply phenotyped Genetic-Phenotype Relationship in Sarcoidosis (GenPhenReSa) cohort of 31 European centers in 12 countries with 116 potentially disease-relevant single-nucleotide polymorphisms (SNPs). Using a meta-analysis, we investigated the association of relevant phenotypes (acute vs. sub-acute onset, phenotypes of organ involvement, specific organ involvements, and specific symptoms) with genetic markers. Subgroups were built on the basis of geographical, clinical and hospital provision considerations.

**Results:**

In the meta-analysis of the full cohort, there was no significant genetic association with any considered phenotype after correcting for multiple testing. In the largest sub-cohort (Serbia), we confirmed the known association of acute onset with TNF and reported a new association of acute onset an HLA polymorphism. Multi-locus models with sets of three SNPs in different genes showed strong associations with the acute onset phenotype in Serbia and Lublin (Poland) demonstrating potential region-specific genetic links with clinical features, including recently described phenotypes of organ involvement.

**Discussion:**

The observed associations between genetic variants and sarcoidosis phenotypes in subgroups suggest that gene–environment-interactions may influence the clinical phenotype. In addition, we show that two different sets of genetic variants are permissive for the same phenotype of acute disease only in two geographic subcohorts pointing to interactions of genetic signatures with different local environmental factors. Our results represent an important step towards understanding the genetic architecture of sarcoidosis.

## Introduction

Sarcoidosis is a clinically heterogeneous disease, particularly regarding the affected organs, the type of onset and the course of the disease (1) and this clinical heterogeneity needs to be addressed therapeutically (2). Sarcoidosis is a complex disorder with a strong genetic background (3–5), for which a number of genetic risk factors have been identified by genome-wide association and candidate gene studies, e.g., various *HLA*-haplotypes (6), *BTNL2* (7), *ANXA11* (8), and *IL23R* (9), some of which confer susceptibility to certain sarcoidosis phenotypes (10). Sarcoidosis is, therefore, pathogenetically regarded as a disease resulting from a complex interplay of an unknown environmental causative agent or agents with a genetically permissive subject. Thus, genetic factors might have a potential to distinguish sarcoidosis phenotypes and should, in general, be most suitable as prospective biomarkers.

Many of the reported genetic associations with sarcoidosis or with a specific clinical phenotype of sarcoidosis could not be replicated in independent studies or showed contradictory results in a replication cohort (4). As an example, the combination of HLA-DRB1*01 and *TNFA2* predisposes Europeans to favorable prognosis, but is associated with poor prognosis and cardiac manifestation of sarcoidosis in a Japanese cohort (11, 12). Similarly, two *ANXA11* variants are associated with sarcoidosis in African Americans, but not in European Americans (13). In some studies, insufficient statistical power due to small sample size could be a reason for this lack of replication. However, in other cases, this might reflect the fact that different causative agents encounter a different genetic architecture to cause sarcoidosis.

The aim of this study was to investigate whether genetic variants known or suggested to be associated with sarcoidosis or implicated in sarcoidosis pathogenesis, are associated with clinical phenotypes of sarcoidosis, including type of onset, phenotypes of organ involvement, specific organ involvements itself, or symptoms and to evaluate whether these associations are consistent throughout multiple European subcohorts or subcohort-specific.

## Patients and methods

### Patient recruitment and phenotyping

The Genetic-Phenotype Relationship in Sarcoidosis (GenPhenReSa) cohort includes a total of 2,163 patients from 31 European centers from 12 countries that were deeply phenotyped at three different time points over a course of 4 years (14). A positive vote by the Ethics Committee of the University of Freiburg was obtained prior to the initiation of the study, which was also registered in the German Clinical Trials Register (identifier DRKS00000045). In short, sarcoidosis patients were recruited to the study who had been diagnosed with sarcoidosis according to the ATS/ERS/WASOG consensus statement (15) with a documented course of disease over at least 2 years prior to and 2 years following recruitment. As a prevention of migration bias only patients from families living for at least 3 generations in the same country were recruited. The 31 centers were grouped into 13 geographic regions based on geographic (16), clinical, hospital provision and sample size considerations. In general, centers were merged to form presumably homogenous regions with sample sizes of at least 24 patients per region. Thus, some countries had two or three geographically distinct regions (Poland with Lublin (*n* = 111) and Gdansk (*n* = 59), and Germany with North (*n* = 161), Central (*n* = 152), and South (*n* = 70)), while small centers from different countries were considered one region based on geographic proximity, e.g., Louven (*n* = 17) and Bobigny (*n* = 8) were merged with Amsterdam (*n* = 67) and Lille (*n* = 31) to form a Netherlands-Belgium-France region. A full list of recruitment centers and regional groups is given in the Supplementary material, and a detailed description of the GenPhenReSa cohort is given elsewhere (14). For this study, data from 1909 patients with complete information on basic variables (age, sex and region of origin) and documentation of the clinical course over 4 years were analyzed. Moreover, clinical and specific organ involvements such as central nervous system (CNS), skin or ocular involvement, and specific symptoms like night sweat, subfebrile temperature, and dyspnoea were collected using the definition of extrapulmonary organ involvement of the ACCESS study (17). Based on the course of disease in the 2 years prior to recruitment, patients were classified as acute or sub-acute onset phenotype (18). Except for this, all phenotypes used for analysis were defined based on the presentation at baseline, i.e., time of recruitment Patients were grouped into five previously described phenotypes of organ involvement (an abdominal, an ocular–cardio–cutaneous–CNS, musculoskeletal–cutaneous, a pulmonary–lymphonodal and an extrapulmonary phenotype) (14, 19).

### Sample and data preparation

DNA from blood samples was isolated using the Qiagen Kit following the manufacturer’s instructions. The amount and quality of all DNA samples was checked by agarose gel electrophoresis, and the DNA was amplified using the REPLI-g whole genome amplification kit prior to genotyping, which was performed using Sequenom Mass-ARRAY iPlex (Sequenom, Inc.) or Taqman (Thermo Fisher Scientific Inc.) technology. Initially, 137 SNPs were selected for investigation based on (i) a known or suggested association with sarcoidosis, (ii) an implication of the gene product in sarcoidosis pathogenesis or (iii) an association of the variant with a clinical phenotype. Allele calling was checked by visual inspection of cluster plots. SNPs or variants with a call rate (CR) < 90%, a minor allele frequency (MAF) < 1% (over all samples from all centers) or an extreme deviation from Hardy–Weinberg equilibrium (*p* < 10^−5^) were excluded from analysis, leaving 116 SNPs for further statistical analysis. A full list of SNPs that were included in the analysis before filtering for these quality control parameters together with references is given in the Supplementary material.

### Statistical methods

All evaluated phenotype variables were dichotomous (present or not), and their frequencies between study regions were compared by chi-square tests. As the main analysis, the overall association between geno- and phenotypes was investigated in the full cohort. Thus, sarcoidosis patients with a specific phenotype were compared to those without the specific phenotype. Taking the nested data structure into account, a meta-analysis using individual participant data (IPD) approach (20, 21) was conducted, where the regional SNP-wise impact upon each phenotype was estimated by a logistic regression model and combined afterwards across all regions to an overall odds ratio (OR). Such a meta-analysis is necessary due to both clinical and genetic heterogeneity between regions (see results section) which could lead to spurious associations in a “mega-analysis” of simple pooled data (Simpson’s paradox). In detail, for each region i (i = 1, …, 13), all SNPs were modelled separately as an independent variable using an additive genetic model (which has good power even under different true genetic models)In all these region-specific logistic regression models, sex, age and smoking status were included as possible confounders such that the resulting OR_i_ are adjusted for these variables. For the meta-analysis, these individually calculated OR_i_, by phenotype and by region, were combined across the geographical regions in a weighted random effects model to get an overall OR, respectively for all phenotypes (22). Low phenotype prevalence and low MAF lead to very imprecise OR estimates and extremely large confidence intervals which cannot sensibly be included in a meta-analysis. However, this depends on sample size and in order to include as much information as possible in the meta-analysis, the region-specific ORs were only excluded from the meta-analysis (and further analyses) if the region-specific combination of phenotype prevalence, SNP MAF and sample size was insufficient to allow a reliable estimation of the OR_i_, i.e., lead to a width of the confidence interval more than 100. Reported ORs always refer to the minor allele: in case of an OR > 1, the minor allele increases the risk of the respective phenotype, while an OR < 1 indicates a reduced risk of the phenotype for the minor allele. Using Bonferroni correction for multiple testing, the significance level of 0.05 was divided by the number of tested SNPs which results in α* = 0.00043 for each phenotype and each SNP in the meta-analysis. Since the considered phenotypes are correlated, we do not correct for the number of phenotypes here.

In an explorative manner, the larger regional sub-cohorts (*n* > 100) were investigated individually in multi-locus models. In particular, for the acute onset phenotype, SNPs with univariate OR which had a *p* value smaller than 0.01 were included in a multiple model. The subsequent model selection was done by likelihood ratio criteria to identify a multi-locus model, which allowed interpretation as a genetic signature. In the explorative analyses of the sub-cohorts, all results with a nominal *p* < 0.01 are presented to balance power and false positive results. Point estimates of the OR are provided with 95% confidence intervals (given in square brackets). All statistical analyses were conducted in R version 3.62 (particularly library metafor (22)).

## Results

### Patient characteristics

In total, 1909 patients from 13 European regions with a minimum number of 24 patients (in Romania) were included in this analysis. Table 1 lists the phenotypes used for the association analysis together with their prevalences in the largest cohorts. The other 323 patients from in total seven regions with a sample size of less than 100 patients each (Romania, *n* = 24, Czech Republic, *n* = 60, Dublin, *n* = 29, England, *n* = 29, Gdansk (Poland), *n* = 59, Iceland, *n* = 52, South Germany, *n* = 70) are here summarized in one separate category and are given in (Supplementary Table 2, see https://www.uni-kiel.de/medinfo/mitarbeiter/freitag-wolf/download/). Frequencies of clinical manifestations (i.e., phenotypes) were significantly heterogeneous between regions (*p* < 0.006 for all characteristics), e.g., in total 755 of 1866 patients suffered from an acute disease onset (40.5%), which had the highest prevalence in the Serbian cohort with 53.6% (480/913) and the lowest prevalence in North Germany with only 18.4% (25/161). In addition to this, the occurrence of missing values differed depending on phenotype and region (23). Genetic heterogeneity, i.e., different minor allele frequencies (see (Supplementary Table 1, for details), between regions was also large, e.g., for rs4143332 at *HLA-B* the minor allele frequency was 7% in the Netherland-Belgian-France region (*n* = 125) and 16% in Serbia (*n* = 913), with even more extreme values in regions with smaller sample sizes, which required the statistical meta-analysis strategy as described above.

**Table 1:** Frequencies (%) of phenotypes together with the proportion of missing values (%M) in total and in the largest subgroups. Neth-Bel-France: the Netherlands-Belgium-France.

Phenotype/Variable	All (*n* = 1,909)		Serbia (*n* = 913)		Northern Germany (*n* = 161)		Central Germany (*n* = 152)		Neth-Bel-France (*n* = 125)		Italy (*n* = 124)		Lublin (Poland) (*n* = 111)		Others (*n* = 323)	
	*N* (%)	%M	*N* (%)	%M	*N* (%)	%M	*N* (%)	%M	*N* (%)	%M	*N* (%)	%M	*N* (%)	%M	*N* (%)	%M
Abdominal	116 (6.9)	11.4	35 (4.9)	21.1	10 (6.5)	3.7	11 (7.5)	3.3	9 (7.2)	0.0	8 (7.0)	8.1	7 (6.3)	0.0	36 (11.3)	1.2
Acute Onset	755 (40.5)	2.25	480 (52.6)	0.1	25 (18.4)	15.5	39 (25.7)	0.0	24 (19.8)	3.2	26 (22.0)	4.8	52 (47.7)	1.8	109 (34.3)	1.5
CNS Involvement	64 (3.4)	0.9	64 (3.4)	0.9	4 (3.2)	0.0	41 (4.5)	0.0	3 (2.8)	1.8	2 (1.3)	0.0	0 (0.0)	0.8	5 (3.2)	3.7
Dyspnea	715 (43.7)	14.3	315 (34.7)	0.5	63 (64.3)	39.1	72 (60.5)	21.7	64 (66.0)	22.4	20 (32.8)	50.8	60 (66.7)	18.9	121 (46.0)	18.6
Extrapulmonary	92 (5.4)	11.4	0 (0.0)	21.1	9 (5.8)	3.7	4 (2.7)	3.3	4 (3.2)	0.0	29 (25.4)	8.1	9 (8.1)	0.0	37 (11.6)	1.2
Eye Involvement	141 (7.5)	1.8	70 (7.7)	0.0	12 (7.9)	6.2	2 (1.3)	2.0	8 (6.4)	0.0	6 (4.9)	0.8	20 (20.0)	9.9	23 (7.3)	2.8
Fever/Subfebrile Temperature	318 (20.8)	19.8	190 (20.9)	0.4	6 (9.8)	62.1	12 (12.1)	34.9	8 (11.0)	41.6	11 (18.3)	51.6	12 (17.1)	36.9	79 (30.5)	19.8
Musculo-skeletal-cutaneous	150 (8.9)	11.4	8 (1.1)	21.1	13 (8.4)	3.7	37 (25.2)	3.3	4 (3.2)	0.0)	12 (10.5)	8.1	28 (25.2)	(0.0)	48 (15.0)	1.2
Night Sweat	189 (12.4)	20.0	89 (9.8)	0.7	4 (6.6)	62.1	21 (20.8)	33.6	2 (2.9)	44.0	0 (0.0)	51.6	19 (26.0)	34.2	54 (21.2)	21.1
Ocular-cardio-cutaneous-CNS	209 (12.4)	11.4	125 (17.4)	21.1	17 (11.0)	3.7	3 (2.0)	3.3	19 (15.2)	0.0	6 (5.3)	8.1	19 (17.1)	0.0	20 (6.3)	1.2
Pulmonary-lymphonodal	1,124 (66.5)	11.4	552 (76.7)	21.1	106 (68.4)	3.7	92 (62.6)	3.3	89 (71.2)	0.0	59 (51.8)	8.1	48 (43.2)	0.0	178 (55.8)	1.2
Skin Involvement	298 (15.7)	0.8	96 (10.5)	0.0	25 (15.9)	2.5	28 (18.4)	0.0	17 (13.7)	0.8	17 (13.7)	0.0	44 (41.1)	3.6	71 (22.4)	1.9

#### Individual participant data meta-analyses

**Figure 1:**
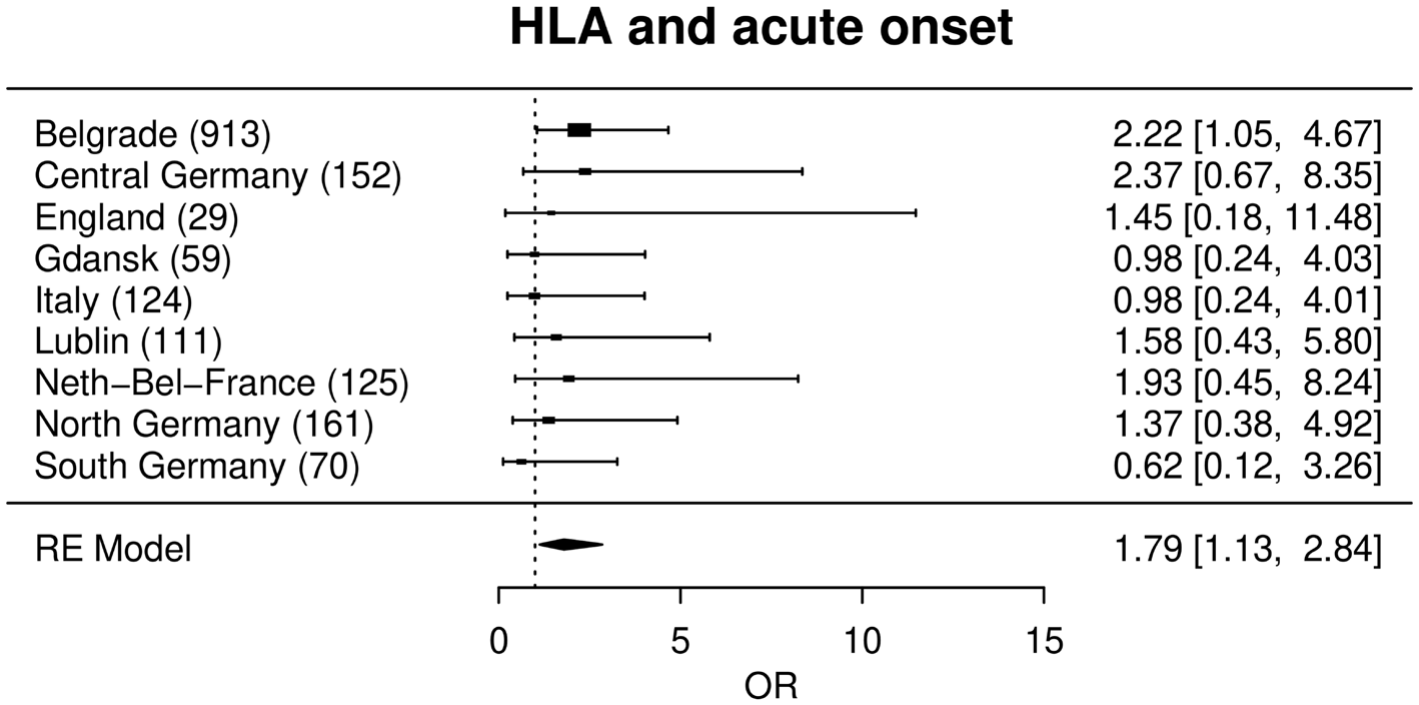
Forest plot with a random effects meta-analysis model for the association between rs4143332 (HLA) and acute onset. Not included are Bucharest, Dublin, Czech Republic and Iceland due to a combination of low sample size, low MAF and low prevalence of acute onset (see statistical methods). Odds ratios (OR) are given with corresponding 95% confidence intervals in square brackets.

In a meta-analysis approach all region-wise effects of each SNP upon the phenotypes were combined for the main analysis. After correction for multiple testing, this analysis did not yield any significant association of any of the investigated genetic variants to any phenotype (Supplementary Table 3, see https://www.uni-kiel.de/medinfo/mitarbeiter/freitag-wolf/download/). The largest effect was found for rs4143332 at *HLA-B* associated with acute onset adjusted for sex, age and smoking status (OR = 1.79, *p* = 0.013), followed by rs1800629 at *TNF* (OR = 1.65, *p* = 0.017), Figures 1 2.

**Figure 2:**
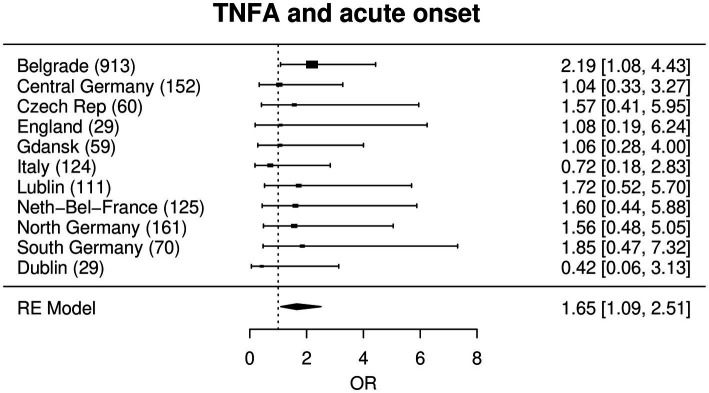
Forest plot with a random effects meta-analysis model for the association between rs1800629 (=*TNFA* -308G/A) and acute onset. Not included are Bucharest and Iceland due to a combination of low sample size, low MAF and low prevalence of acute onset (see statistical methods). Odds ratios (OR) are given with corresponding 95% confidence intervals in square brackets.

Focusing on and summarizing the Central Europe regions (Germany and Netherlands-Belgium-France), the estimated effects were slightly smaller for the above mentioned two loci (OR = 1.58, *p* = 0.19 and OR = 1.42, *p* = 0.27, respectively). As an aside, the association with the smallest *p*-value in the pooled meta-analysis of the Central Europe regions was night sweat and rs2075800, a missense variant near *HSPA1L* (OR = 3.95, *p* = 0.0075).

#### Region-specific analyses

**Figure 3:**
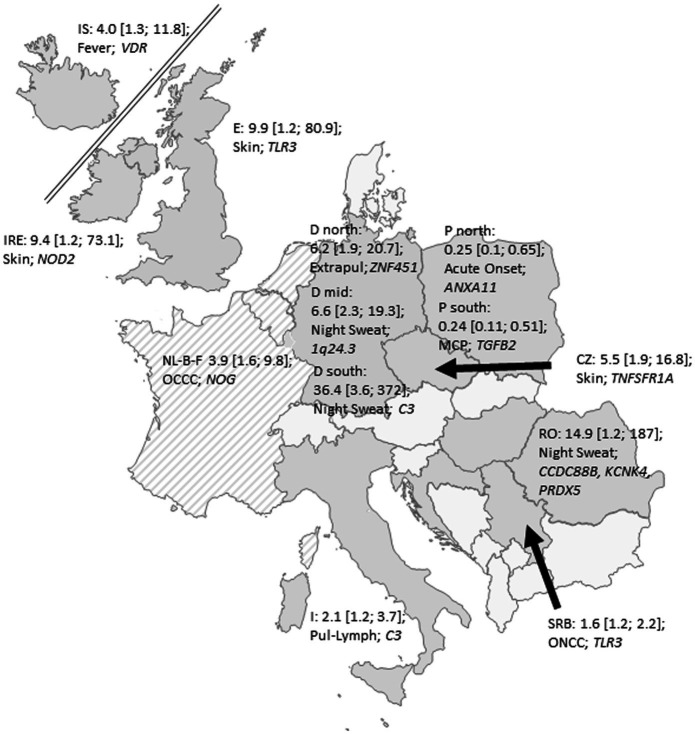
Map of the recruited European countries with their most relevant phenotype–genotype relation (smallest *p*-values) for each of the analyzed 13 regions expressed as OR with 95% confidence intervals; IS: Iceland; I: Italy; IRE: Ireland; E: England; D north: Northern Germany; D mid: Central Germany; D south: Southern Germany; NL-B-F: the Netherlands-Belgium-France Neth-Bel-France; I: Italy; SRB: Serbia; RO: Romania: Pl1: Gdansk, Poland: Pl2; Lublin, Poland CZ: Czech Republic; Pul-Lymph: Pulmonary–Lymphonodal Phenotype; ONCC: Ocular–Cardio–Cutaneous–CNS Phenotype; Extrapul: Extrapulmonary Phenotype; MCP: Musculoskeletal–Cutaneous Phenotype. Grey: participating countries; hatched: pooled cohorts.

Because of the strong phenotypic and genetic heterogeneity, we also report results of the largest individual sub-cohorts as exploratory analyses. We investigated potential genetic associations with type of onset, phenotypes of organ involvement, specific organ involvements and symptoms within the defined regional sub-cohorts. Apart from acute onset, none of the tested phenotypes in the regions reached the required level of significance after correcting for multiple testing and all reported *p* values in the subsequent paragraphs are nominal p values. Figure 3 gives a map of the 12 included European countries with their most relevant phenotype–genotype relation in terms of their smallest *p*-values for each of the analyzed 13 regions. All potentially meaningful (*p* < 0.01) ORs, CI and p-values of all regions are given in Table 2 and all results with *p* < 0.05 are given in Supplementary Table 3. In the following, we report details on associations with *p* < 0.01.

**Table 2:** Single locus region-specific ORs (sex-, age, and smoking-status adjusted) for phenotypes with *p* values < 0.01. OR: Odds Ratio; CI: 95% confidence interval.

Subgroup	Phenotype	Gene	SNP	OR [95% CI]	*p*
Central Germany	Abdominal phenotype	*NFKBIA*	rs2233409	5.56 [1.69; 16.67]	0.004
	Abdominal phenotype	*ITGAE/NCBP3*	rs2891	5.26 [1.63; 16.67]	0.005
	Dyspnoea	*1q24.3*	rs12035082	2.28 [1.21; 4.25]	0.0098
	Dyspnoea	*ACE*	rs4342	2.32 [1.26; 4.34]	0.008
	Fever or sub-febrile temperatures	*HMOX1*	rs2071746	4.16 [1.41; 11.1]	0.0096
	Night sweat	*1q24.3*	rs12035082	6.61 [2.29; 19.05]	0.0005
	Night sweat	*HLA-DRB1/BRB5*	rs9378264	3.17 [1.48; 6.82]	0.003
	Night sweat	*KDR*	rs7667298	3.39 [1.49; 7.73]	0.004
North Germany	Dyspnoea	*IL23R*	rs7517847	3.32 [1.52; 7.23]	0.003
	Dyspnoea	*SLC11A1*	rs2279015	2.5 [1.25; 5.0]	0.0095
	Extrapulmonary phenotype	*RAB23*	rs3800018	6.51 [1.96; 21.63]	0.002
	Extrapulmonary phenotype	*CCL18*	rs2015086	4.51 [1.72; 11.85]	0.002
	extrapulmonary phenotype	*ZNF451*	rs7756421	6.53 [1.99; 21.41]	0.002
	Fever or sub-febrile temperatures	*IL10*	rs3024505	33.65 [2.87; 394.6]	0.005
Serbian Cohort	Abdominal phenotype	*BTNL2*	rs5007259	2.13 [1.31; 3.70]	0.003
	Abdominal phenotype	*IL1RN*	rs454078	2.05 [1.23; 3.43]	0.006
	Acute Onset	*TNFA -308G/A*	rs1800629	2.19 [1.70; 2.82]	1.31 × 10–9
	Acute Onset	*HLA-B*	rs4143332	2.22 [1.67; 2.94]	3.60 × 10–8
	Acute Onset	*HLA-DQA1*	rs9271366	1.69 [1.24; 2.30]	9.63 × 10–4
	Dyspnoea	*ANXA11*	rs1049550	1.36 [1.09; 1.69]	0.006
	dyspnoea	*IL10*	rs3024505	1.51 [1.12; 2.04]	0.007
	Fever or sub-febrile temperatures	*NFKBIA*	rs2233409	1.58 [1.15; 2.17]	0.005
	Neurosarcoidosis	*TLR3*	rs3775291	2.01 [1.27; 3.16]	0.003
	Neurosarcoidosis	*IL12B*	rs4921492	1.88 [1.18; 2.99]	0.008
	Neurosarcoidosis	*LOC102723568*	rs12793173	1.89 [1.20; 3.03]	0.006
	Ocular–cardio–cutaneous–CNS	*LOC102723568*	rs12793173	1.47 [1.11; 1.96]	0.006
	Ocular–cardio–cutaneous–CNS	*TLR3*	rs3775291	1.59 [1.20; 2.11]	0.001
	Ocular sarcoidosis	*PTGS2*	rs2206593	2.20 [1.21; 4.0]	0.0096
	Ocular sarcoidosis	*IL18*	rs1946518	1.61 [1.14; 2.26]	0.006
	Ocular sarcoidosis	*IL18*	rs189667	1.69 [1.23; 2.44]	0.004
	Skin involvement	*SPP1*	rs11730582	1.59 [1.16; 2.17]	0.004
	Skin involvement	*TNF*	rs1800629	1.82 [1.18; 2.86]	0.008
	Skin involvement	*6p21*	rs4143332	2.22 [1.27; 3.85]	0.005

#### Acute vs. sub-acute onset

In the Serbian sub-cohort (Table 2), the strongest association of acute onset in the single locus model was found for rs1800629 (=*TNFA*-308G/A), a well-known sarcoidosis risk SNP that is located in the *TNF* promoter region: the A-allele was significantly associated with acute onset of the disease with an OR of 2.19 and displayed the smallest p value in the regression analysis (*p* = 1.31 × 10^−9^*).* Moreover, one marker in the HLA-region showed a significant association, namely rs4143332 in the *HLA-B* gene region (*p* = 3.60 × 10^−8^; OR = 2.22). The next strongest signal was found with rs9271366 near *HLA-DQA1* (*p* = 9.63 × 10^−4^; OR = 1.69; (Supplementary Table 3).

#### Phenotypes, organ involvement and symptoms

We recently defined novel phenotypes of organ involvement: an abdominal, an ocular–cardio–cutaneous–CNS, a musculoskeletal–cutaneous, a pulmonary–lymphonodal and an extrapulmonary phenotype (14). Here, we analyzed whether these phenotypes are associated with genetic variants and even though none of these reached statistical significance after adjustment for multiple testing, several are still noteworthy with large effect sizes in individual sub-cohorts (Supplementary Table 3). The extrapulmonary phenotype was associated with rs2015086 near *CCL18* (*p* = 0.002; OR = 4.51), rs3800018 near *RAB23* (p = 0.002; OR = 6.51) and rs7756421 near *ZNF451* (p = 0.002; OR = 6.53) in the North German sub-cohort. The abdominal phenotype was associated with rs5007259 at *BTNL2* (*p* = 0.003; OR = 0.47) and rs454078 *IL1RN* (*p* = 0.006; OR = 2.05) in the Serbian sub-cohort, and with rs2233409 near *NFKBIA* (*p* = 0.004; OR = 0.18) and rs2891 near *ITGAE/NCBP3* (*p* = 0.005; OR = 0.19) in the Central German sub-cohort. The ocular–cardio–cutaneous–CNS phenotype showed associations with rs12793173 near *LOC102723568* (*p* = 0.006; OR = 0.68) and rs3775291 in the *TLR3* gene – resulting in a missense variant - (*p* = 0.001; OR = 1.59) in the Serbian sub-cohort.

#### Specific organ involvements

Sarcoidosis is a highly variable disease, especially variable in the distribution of affected organs. We hypothesized that specific genetic variants are associated with distinct organ involvements in sarcoidosis. In the Serbian sub-cohort we found noteworthy risk loci for neurosarcoidosis and ocular sarcoidosis. The minor alleles at three genetic variants are associated with increased risk of neurosarcoidosis: rs3775291 near *TLR3* (*p* = 0.003; OR = 2.01), rs4921492 near *IL12B* (*p* = 0.008; OR = 1.88), while the minor allele at rs12793173 near *LOC102723568* (p = 0.006; OR = 0.53) is protective. The following risk loci were observed for ocular sarcoidosis: rs2206593 near *PTGS2* (*p* = 0.0096; OR = 2.20), and two variants near *IL18*, however with opposite directionality: rs1946518 (p = 0.006; OR = 1.61) and rs189667 (*p* = 0.004; OR = 0.59). In the Serbian cohort, skin involvement was associated with rs11730582 near *SPP1* (p = 0.004; OR = 1.59), the aforementioned *TNF* variant rs1800629 (*p* = 0.008; OR = 0.55) and rs4143332 at 6p21 (*p* = 0.005; OR = 0.45). Again, borderline significance of these associations prevents any definite conclusions without further confirmation in other cohorts.

#### Symptoms

Next, we analyzed whether specific symptoms in patients with sarcoidosis are influenced by distinct genetic variants. This analysis might give valuable hints towards the underlying, genetically influenced bodily response to the disease. In the Central German sub-cohort, night sweat was associated with rs12035082 at 1q24.3 (*p* = 0.0005; OR = 6.61), rs9378264 near *HLA-DRB1/BRB5* (*p* = 0.003; OR = 3.17) and rs7667298 near *KDR* (p = 0.004; OR = 3.39). We identified novel genetic associations for fever or sub-febrile temperatures with rs3024505 near *IL10* (p = 0.005; OR = 33.65) in the North German sarcoidosis patients, rs2233409 near *NFKBIA* (p = 0.005; OR = 1.58) in the Serbian patients, and rs2071746 near *HMOX1* (p = 0.0096; OR = 0.24) in patients from Central Germany. Dyspnoea in sarcoidosis patients was associated with rs1049550 near *ANXA11* (*p* = 0.006; OR = 1.36) and rs3024505 near *IL10* (*p* = 0.007; OR = 0.66) in the Serbian cohort, rs12035082 at 1q24.3 (*p* = 0.0098; OR = 2.28) and rs4342 near *ACE* (p = 0.008; OR = 0.43) in the Central German cohort, and rs7517847 near *IL23R* (p = 0.003; OR = 3.32) and rs2279015 near *SLC11A1* (*p* = 0.0095; OR = 0.4) in the North German cohort. Taken together, these results suggest that the emergence of some symptoms in patients with sarcoidosis might indeed be associated with specific genetic loci, however, no association reached statistical significance when adjusted for multiple testing.

#### Multi-locus models

In the Serbian and Lublin (Poland) cohorts, two separate sets of three SNPs in six different genes showed association to the acute onset phenotype in multi-locus models (Table 3). In the Serbian cohort, after backward selection of all loci with *p* < 0.01, the individual impact of the analyzed SNPs on acute onset decreased for rs1800629 from OR = 2.19 in the single-locus model to 1.95 in the multi-locus model and for rs9271366 from OR = 1.69 to OR = 1.43, whereas the OR of rs4143332 increased slightly from 2.22 to OR = 2.34. A combination of the risk alleles at all three SNPs conferred an OR of 6.6 when considered as a genetic signature. In Lublin, all three SNPs had substantially stronger effects in a multi-locus model and the combined effect of a genetic signature comprising all three risk alleles corresponded to an OR of 37.9. Two of the SNPs in this genetic signature, namely rs1891467 near *TGFB2* and rs7667298 near *KDR,* had been reported to be associated with an acute course of sarcoidosis before in a German cohort (4).

**Table 3:** Single and multi-locus models of acute onset in the Serbian and Lublin (Poland) sub-cohort. The ORs are sex-, age, and smoking-status adjusted. OR: Odds Ratio; CI: confidence interval.

Origin	SNP	Locus	Alleles	Single-locus univariable model		Multi-locus multivariable model		Genetic signature
				OR [95% CI]	*p*	OR [95% CI]	*p*	OR
Serbia	rs1800629	*TNF*	G > A	2.19 [1.70;2.81]	1.31 × 10^−9^	1.95 [1.33; 2.89]	0.001	}6.6
	rs4143332	*HLA-B*	G > A	2.22 [1.67;2.94]	3.6 × 10^−8^	2.37 [1.26; 4.54]	0.008	
	rs9271366	*HLA-DR–DQ*	G > A	1.69 [1.24;2.30]	0.001	1.43 [1.05; 1.95]	0.023	
Poland (Lublin)	rs1148459	*TNFRSF1B*	G > T	2.30 [1.29; 4.10]	0.005	3.16 [1.59; 6.29]	0.001	}37.9
	rs1891467	*TGFB2*	G > A	3.41 [1.47; 7.91]	0.004	4.56 [1.75; 11.90]	0.002	
	rs7667298	*KDR*	C > T	0.56 [0.32; 1.00]	0.049	0.38 [0.19; 0.76]	0.006	

In amalgamate, two different sets of genetic variants are permissive for the same phenotype of acute disease onset resulting in combined ORs of 6.6 for the Serbian and of 37.9 for the Polish subcohort which points at their likely interactions with different local environmental factors resulting in the same phenotype.

## Discussion

Sarcoidosis is a highly variable and heterogeneous disease with a need for understanding the genetic background of this heterogeneity. In total 116 selected candidate SNPs were analyzed for their association with clinical traits in sarcoidosis patients of the GenPhenReSa cohort (14). The GenPhenReSa patient collection represents a large sarcoidosis cohort of almost 2,000 patients of European origin that was deeply phenotyped over 4 years at three points in time, i.e., every 2 years (14). In line with the known heterogeneity, we did not observe any genotype–phenotype association that was uniformly present in the whole European cohort, when analyzed with an appropriate meta-analysis. However, different unique associations were present in different geographic sub-cohorts. With our approach, we were able to identify specific SNPs that are associated with course of the disease (acute onset) in the Serbian and Lublin (Poland) sub-cohorts which result in increased ORs if used as a set. In addition, we found some genetic associations with the phenotypes of organ involvement and specific symptoms, mostly restricted to individual geographic cohorts (Figure 3). In these smaller samples, effect sizes were large but did not reach the predefined, stringent significance levels.

Most strikingly and of potential prognostic value, marker rs1800629 (=*TNF*-308G/A), a SNP in the *TNF* promoter region, was associated with acute onset. The A-allele of this SNP is associated with acute onset with an OR of 2.18 in the Serbian sub-cohort, which is in line with previous reports that describe the A-allele being associated with a favorable prognosis (24). Likewise, associations with acute onset were observed for the HLA variants rs4143332 (OR = 2.22) and rs9271366 (OR = 1.69). The latter variant is located between *HLA-DRB1* and *HLA-DQA1* and is associated with inflammatory bowel disease, multiple sclerosis, systemic lupus erythematosus and others (25–27), but has not yet been reported with regards to sarcoidosis. Whether or not this finding might be dependent on HLA haplotypes that are known to be associated with certain sarcoidosis subtypes has to be analyzed in future studies. Noteworthy, used in combination, these variants are associated with acute onset sarcoidosis with an OR of 6.6 (Table 3) which could be used as a genetic signature.

Interestingly, the estimated effects of SNPs near *TNFRSF1B*, *TGFB2*, and *KDR* on acute onset in the Polish sub-cohort from Lublin increased in the multi-locus models and result in an OR of 37.9 (Table 3) when considered as a genetic signature. *TGFB2* and *KDR* variants were reported to be associated in a German cohort with an acute course of sarcoidosis (4) and variants of *TNFRSF1B* are known to influence the response to anti-TNF therapy in sarcoidosis (28). Which leads to the concept of a possible regulation of acute sarcoid inflammation by these genetic variants.

The effects of SNPs near *TNFSFR1B*, *TGFB2* and *KDR* on acute onset are increased in the multi-locus models. The latter two SNPs were reported to be associated with an acute course of sarcoidosis before (4). Different roles of the variant gene products in the regulation of inflammation can be assumed on the basis of *ex-vivo* data. The SNP rs1148459 is located at the position −9,298 of the 5′-flanking region of *TNFRSF1B* and it is speculated that this SNP may regulate the expression of the receptor (29). *TNFSFR1B* is associated with the generation of Treg (30). Lack of *TNFSFR1B* expression of Treg may lead to non-functional Treg which suggests a functional influence on the effect of anti-TNF therapy of sarcoidosis (28). The SNP rs1891467 is located in the beginning of the second intron of *TGFB2,* however, the consequences of the SNP are unclear. Members of the TGFβ family are anti-inflammatory mediators (31) and in bronchoalveolar lavage cell culture supernatants from patients suffering from active sarcoidosis with spontaneous remission we found increased levels of TGFβ indicating an anti-inflammatory activity of TGFβ also in sarcoidosis. However, it is currently not clear whether or not this is related with SNP rs1891467. The SNP rs7667298 is located in the 5’UTR of the gene *KDR* which codes for the VEGF-R2. It has been demonstrated that rs7667298 is related with diminished expression of VEGF-R2 (32). In contrast, in sarcoidosis increased levels of VEGF have been detected (33). VEGF-R2 is a strong inducer of vascularization whereas VEGF-R1 exerts pro-inflammatory stimulation. Thus, lack of VEGF-R2 may lead to abnormal vascularization (33) but fosters inflammation. Thus, relevance of these genetic variants on the up- and down-regulation of sarcoid alveolitis is most likely.

The number of significant associations between SNPs and clinical variables in the entire cohort of 1909 Caucasians fell short of expectations, which may suggest that permissive genetic signatures depend strongly on specific environmental or occupational events in a particular geographic region or cohort to cause a particular phenotype as suggested by the ACCESS-study (19, 34). This conclusion seems likely since all patients came from families that lived for at least three generations in the same region. Nevertheless, the application of genetic markers as predictors for certain phenotypes has turned out to be challenging for the majority of complex diseases and various methods have been applied to identify risk loci sets [reviewed in (35)]. A critical point in our study may have been the choice of markers that were genotyped which had been selected because of previous reports in sarcoidosis or other granulomatous diseases. As this was not a genome-wide association study, we might have missed important genetic associations. Furthermore, an association with the investigated phenotypes had not been established before for the majority of the analyzed SNPs and therefore replication in independent cohorts of diverse ethnicities and with different study designs is warranted.

Within the entire cohort, which comprised a large, genetically and phenotypically heterogeneous population there were no clearly significant genetic associations of the phenotypes of organ involvement; however, the observed effects were large (ORs >2 or <0.5) within specific and more homogeneous geographic areas.

In this multicentre study, the number of patients and prevalences of specific phenotypes for the clinical variables were not large enough to detect significant genetic effects on most phenotypes. Indeed, in terms of statistical considerations, with the overall sample size of more than 1900 patients, an OR larger 1.3 could have been found with a power above 90% (additive model, alpha set to 0.00043, allele frequency assumed to be 0.25), but the genetic and phenotypic heterogeneity of the included patients turned out to be most challenging. While the genotype frequencies of investigated markers show at least a slight gradient within Europe (16), which was addressed by the meta-analysis, the phenotypic heterogeneity was so large that it could not be fully compensated by statistical methods. This large phenotypic heterogeneity between regions might be due to differences in health care systems and recruitment settings (different age distributions of cases, different commonly used treatment) and different frequencies of individual non-genetic risk factors such as smoking which has been reported to increase, e.g., the susceptibility in Sweden by a gene–environment interaction (36). Moreover, about 30% of the sarcoidosis incidence is attributed to occupational exposures (37) and many associations of sarcoidosis with environmental, infectious or occupational exposures have been reported (19, 38, 39). Unfortunately, individual exposure information is not available for the GenPhenReSa cohort. Chronic Beryllium Disease (CBD) is a perfect phenocopy of sarcoidosis and SNPs associated with CBD exert janiform functions depending on disease stage which might also be the case in sarcoidosis which demands more detailed phenotyping in future studies (40). Thus, gene - environment / occupation interactions have to be considered which cause a complex setting of exposures to be analyzed. An example would be the specific T-cell response to a peptide of Aspergillus nidulans documented in Swedish HLA-DR3 positive Löfgren’s syndrome patients (41). Further genetic studies are needed and our data presented in the (Supplementary Tables will be very helpful in designing future studies.

Taken together, we confirmed the association of rs4143332 at *HLA-B* and rs1800629 at *TNF* locus with acute onset of sarcoidosis in the Serbian sub-cohort and describe potential genetic links with several clinical features, including the recently described phenotypes of organ involvement (14, 19). Furthermore, we demonstrate that the concept of genetic signatures needs to be complemented with environmental and occupational data. The observed associations of genetic and clinical features suggest that the clinical phenotype is highly dependent on subcohort-specific environmental interactions with genetics and is more complex than anticipated. This view is supported by the fact that in the Polish and German geographic subcohorts different gene variants exhibited highest ORs (Figure 3). Most likely different genetic backgrounds in combination with local exposures are responsible for identical sarcoid phenotypes in different environmental and regional settings of Europe (14, 19). Our finding represents an important and necessary step towards understanding the genetic architecture of the heterogeneous and complex disease sarcoidosis. Local signatures comprising genetic, occupational, environmental and infectious parameters will be necessary and seem achievable to predict courses of the disease.

## Data availability statement

The datasets presented in this study can be found in online repositories. The names of the repository/repositories and accession number(s) can be found at: https://www.uni-kiel.de/medinfo/mitarbeiter/freitag-wolf/download/.

## Ethics statement

The studies involving human participants were reviewed and approved by Ethik-Kommission der Albert-Ludwigs-Universität Freiburg, email: ekfr@uniklinik-freiburg.de. The patients/participants provided their written informed consent to participate in this study.

## Author contributions

JM-Q, SS, GZ, and AP: design and planning of study, application for funding, directing performance, obtaining ethics committee vote, and clinical trials registration. JCS, RK, VM-V, JM, DJ, EB, PR, DB, RJ, L-PH, KG, AnD, BM, AG, MiP, MK, SH, FB, CGrah, TP-G, ZK, SP, CGrohé, JS, MS, AM, JH, WW, LS, MaP, DV, VP, HW, and AP: patient recruitment, phenotyping, physical examination, radiograph reading, checking inclusion criteria, and documentation. AF and GZ: biobanking and DNA quality control. AP, JM-Q, JCS, EB, and PR: phenotyping quality control. JM-Q, JS, and BF: clinical data quality control. SS, AF, and RA: genotyping. SF-W, SS, and AsD: genotyping quality control. SF-W, AsD, JCS, SS, RA, JM-Q, and BF: data analysis. SF-W and AsD: statistics. SF-W, AsD, JCS, JM-Q, BF, and GZ: manuscript writing. All authors contributed to the article and approved the submitted version.

## Funding

This study was supported by German Federal Ministry for Education and Research grant 01EY1103 and German Research Foundation grant MU 692/8-1 and FI 1935/1-1, DFG Exzellenzcluster PMI EXC 2/67 and Clusterlab XI EXC 306/2. JCS is supported by the CORE100Pilot Advanced Clinician Scientist Program of Hannover Medical School funded by Else Kröner-Fresenius Foundation (2020_EKSP.78) and the Ministry of Science and Culture of Lower Saxony, and the Fritz Thyssen-Foundation.

## Conflict of interest

FB received from Boehringer Ingelheim, Fujirebio, Galapagos NV and Roche; personal travel support from Boehringer Ingelheim and Roche, board membership fees from Boehringer Ingelheim, Bristol Myers Squibb, Fujirebio, Galapagos, GlaxoSmithKline, Roche and Takeda. DB grants paid to the institution from Synairgen and Sanofi; personal honoraria received from Chiesi, Norvatis and Sanofi; personal honoraria for lectures received from Astra Zeneca, Eli Lilly and Norvatis; not for profit activities for ministry of health, Romania. AF received a grant from the German Research Foundation. BF: received grants from Bristol Myers Squibb and AdVita Lifescience paid to the institution; personal honoraria for lectures from Boehringer Ingelheim, Astra Zeneca and Roche, personal travel support from Boehringer Ingelheim; reports a pending patient WO2020225246A1 and stocks from Relief Therapeutics. CG received lecture fees from Astra Zeneca paid to the institution. MK received consulting fees from Boehringer Ingelheim. JM-Q grants from German Research Council, Bristol Myers Squibb and AdVita Lifesciences paid to the institution, received personal honoria from AdVita Lifesciences and Roche, received lecture fees from Astra Zeneca and Roche, received payments for expert testimony from AdVita Lifescience; received travel support from AdVita Lifescience and Grifols, reports a pending patent WO2020225246A1 and stocks from Relief Therapeutics. MaP received grants from Palacky University paid to the institution. MiP reports fiduciary activities for Deutsche Gesellschaft für Pneumologie und Beatmungsmedizin. VP received personal consulting fees from Ambu and Erbe; lecture honoraria from Boehringer Ingelheim and Roche and participated in a data safety monitoring board for Boehringer Ingelheim. AP received personal consulting fees from Boehringer Ingelheim, Roche, AstraZeneca and Galapagos, lecture fees from Boehringer Ingelheim, Norvatis and Gilead, participated in data safety monitoring boards for Boehringer Ingelheim, Roche and AstraZeneca and reports fiduciary activities for the European Rare Disease Network Interstitial Lung Disease Group and the steering committee of the World Association of Sarcoidosis and Other Granulomtous Disorders. SS reports grants from the German Research Council paid to the institution, personal consulting fees from Abbvie, Arenal, BMS, Biogen, Celltrion, Celgen, IMAB, Gilead, MSD, Mylan, Pfizer, Fresinius, Janssen, Takeda, Theravance, Provention Bio, Protagonist, Falk, Ferring, UCB, Amgen, Sandoz Hexal, Lilly, Galapagos. JCS grants from the German Research Council, Else Kröner-Fresenius Foundation, and Fritz Thyssen-Foundation paid to the institution; received personal lecture honoraria from Boehringer Ingelheim. LS received personal honoraria from Boehringer Ingelheim for educational events. D received personal honoraria from Boehringer Ingelheim for activities in advisory boards and lecture fees from Boehringer Ingelheim and AstraZeneca and reports travel support from Boehringer Ingelheim. WW grants from Roche, Boehringer Ingelheim, and Galapagos, consulting fees from Sanofi, Boehringer Ingelheim and Roche, honoraria for lectures from Roche and Beohdringer Ingelheim and payments for activities in data safety monitoring boards from Boehringer Ingelheim and Roche all paid to the institution. GZ reports a pending patent (EP 2585089 A1) and stock options for Moderna, Biontech and Relief Therapeutics.

The remaining authors declare that the research was conducted in the absence of any commercial or financial relationships that could be construed as a potential conflict of interest.

## Publisher’s note

All claims expressed in this article are solely those of the authors and do not necessarily represent those of their affiliated organizations, or those of the publisher, the editors and the reviewers. Any product that may be evaluated in this article, or claim that may be made by its manufacturer, is not guaranteed or endorsed by the publisher.
